# Age is a predictor of elbow stiffness after type III or IV supracondylar humerus fractures

**DOI:** 10.1007/s00590-024-04031-4

**Published:** 2024-06-25

**Authors:** Kavish Gupta, Mary Kate Erdman, Ali Siddiqui, Mathew Schur, Erin Meisel, Rachel Y. Goldstein

**Affiliations:** 1 Cedars-Sinai Medical Center, Los Angeles, CA USA; 2 Department of Orthopaedic Surgery and Rehabilitation Medicine, University of Chicago Medicine and Biological Sciences, Chicago, IL USA; 3 Department of Orthopaedic Surgery and Rehabilitation, University of Florida College of Medicine-Jacksonville, Jacksonville, FL USA; 4 Department of Orthopaedic Surgery, University of Southern California Keck School of Medicine, Los Angeles, CA USA; 5 Children’s Orthopaedic Center, Children’s Hospital Los Angeles, 4650 Sunset Blvd, MS #69, Los Angeles, CA 90027 USA

**Keywords:** Elbow, Pediatric fracture, Outcome, Supracondylar humerus fracture, Age

## Abstract

**Purpose:**

Supracondylar humerus (SCH) fractures account for approximately 30% of injuries for those younger than 7 years of age (Cheng et al. in J Pediatr Orthop 19:344–350, 1999). Recent studies examining the association of patient age and SCH fracture outcomes have provided conflicting findings. The purpose of this study is to investigate SCH fracture outcomes in children at different ages of skeletal development.

**Methods:**

Retrospective review of a Level I pediatric trauma center between 2010 and 2014 was conducted. 190 patients with SCH fractures, age < 14 years, fracture type Gartland III or IV (AO/OTA 13-M 3.1 III and IV) were included. Patients were sorted into age groups: < 2 years, 4–6 years, and > 8 years. Patients were treated with either a closed or open reduction with percutaneous fixation. Clinical outcomes including postoperative elbow range of motion, nerve palsy, compartment syndrome, infection, and cubitus varus were assessed.

**Results:**

Patients in younger age groups were more likely to obtain postoperative full elbow flexion (< 2 years = 77%; 4–6 years = 66%; > 8 years = 43%) and full elbow extension (< 2 years = 96%; 4–6 years = 88%; > 8 years = 64%). Age was a significant predictor of nerve palsy on admission, mean operative time (< 2 years = 21.8 min; 4–6 years = 43.0 min; > 8 years = 80.7 min), and mean fluoroscopy time (< 2 years = 22.9 s; 4–6 years = 59.5 s; > 8 years = 171.9 s). There were no differences in rates of open reduction, compartment syndrome, pin tract infection, cubitus varus, or reoperation among groups.

**Conclusion:**

Increasing age is associated with increased elbow stiffness after percutaneous fixation of Gartland Type III and Type IV SCH fractures. Older patients with SCH fractures may benefit from formal rehabilitation.

**Level of evidence:**

III.

## Introduction

Supracondylar humerus (SCH) fractures are some of the most common injuries in the pediatric population. They account for up to 30% of all fractures in children younger than 7 years old and more than 10,000 emergency department visits annually [1, 2]. While the need for surgery can be controversial for some Gartland type II SCH fractures, most pediatric orthopedic surgeons agree that type III and IV fractures are treated with either closed or open reduction and percutaneous pinning [3]. Immediate short-term concerns include limb threatening complications such as compartment syndrome and disruption of blood supply to the distal upper extremity. At least 10% of type III SCH fractures present with signs of vascular compromise [4, 5]. Additionally, long-term complications such as cubitus varus and restricted elbow range of motion (ROM) can significantly impact function. SCH fractures occur most commonly in children between ages 2 and 7 but can happen in children of any age [2]. Few previous studies have examined the association between patient age and complication rate.

Relevant studies that have been published have provided conflicting findings. In a 2012 retrospective review of 1297 pediatric patients with operative SCH fractures, Fletcher et al. reported patients older than 8 years of age had higher rates of pin tract infection and postoperative stiffness than younger patients [6]. In 2018 Robertson et al. published a review of 31,167 isolated SCH fractures in the National Trauma Data Bank which found older age to be a risk factor for compartment syndrome (OR 1.1; 95% CI 1.0–1.2; *p* < 0.0092) [7]. Contrary to these findings, Mitchelson et al. found that age did not have an effect on complication rates in their retrospective review of 382 SCH fractures [8]. The purpose of this study is to further investigate the effect of age on outcomes of supracondylar humerus fractures.

## Materials and methods

A retrospective review was conducted on patients with SCH fractures who were treated at a single level I pediatric trauma center from 2010 to 2014. Patients aged < 14 years with a Gartland type III or IV SCH fracture (AO/OTA 13-M 3.1 III and IV) [9] who underwent closed or open reduction and percutaneous pinning were included. Medical charts and radiographs were reviewed for demographic information, clinical symptoms, operative report, and complications. Data were collected on presence of a nerve palsy on admission, fracture type, open versus closed reduction, total operative time, fluoroscopy time, reoperation, range of motion at final follow-up, and complications including cubitus varus, compartment syndrome, and pin tract infection. Patients were categorized into age-based groups: < 2 years, 4–6 years, and > 8 years to create 3 distinct study groups and minimize type II error. Differences in characteristics and outcomes were compared across groups.

### Statistical analysis

Statistical analysis was performed using STATA software (version 14.0.371; StataCorp, LLC). Descriptive statistics were used to outline demographic characteristics, complication rates, and incidence of reoperation. Univariate analysis was performed to compare complication rates, rates of reoperation, need for open versus closed reduction, total operative and fluoroscopy time, and rate of return of full elbow flexion and extension at final follow-up. Differences in demographics, operative time, and fluoroscopy were analyzed using analysis of variance, and complications were analyzed using chi-square tests. Significance was defined by a *p* value < 0.05, and all tests were two-tailed. Pairwise comparisons were also made using a Bonferroni adjusted significance level of 0.017.

## Results

**Fig. 1:**
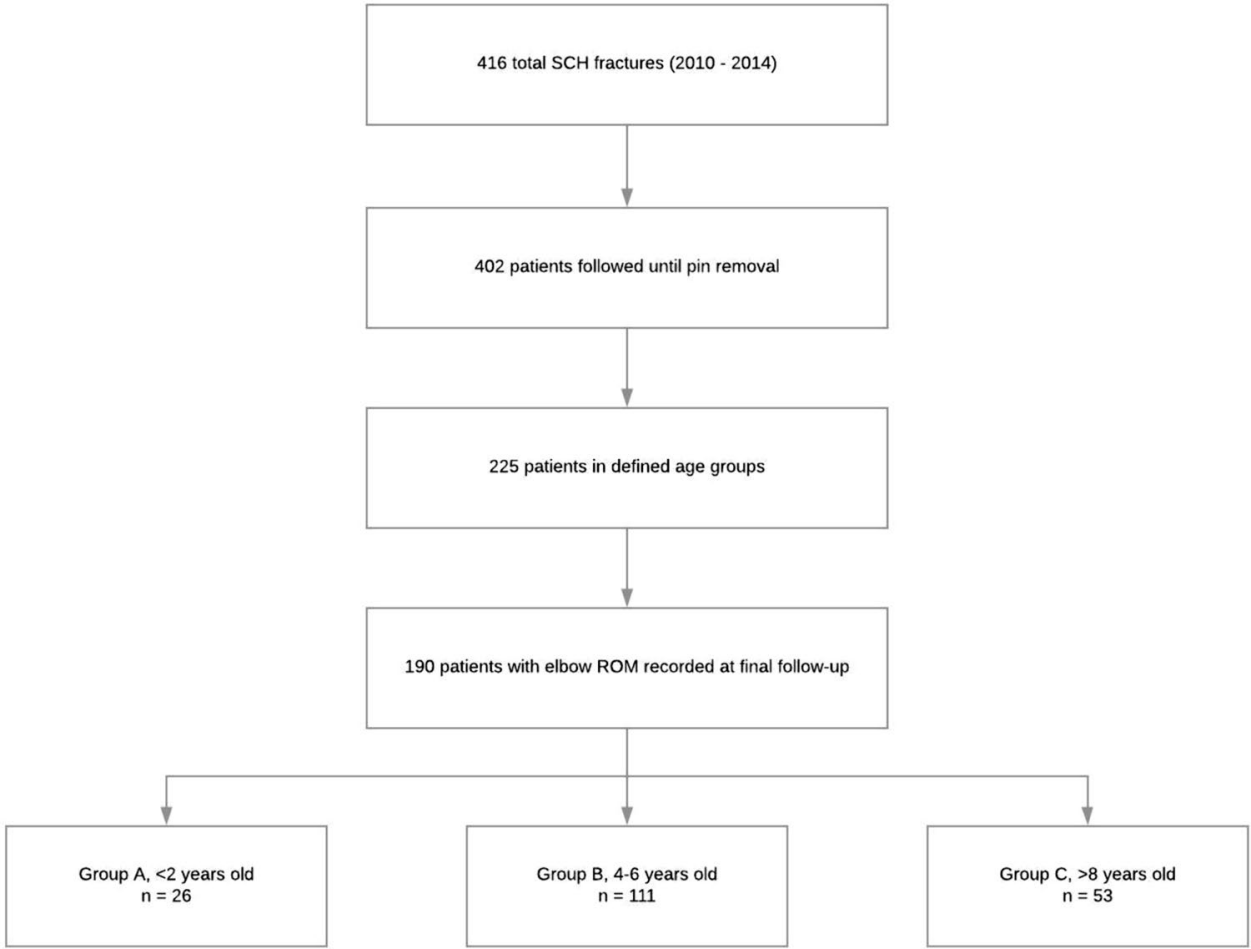
Study design flow diagram

One-hundred and ninety patients with 190 SCH fractures met inclusion criteria (Fig. 1). Mean age was 5.8 ± 2.7 (range 0.2–13) years and mean follow-up 3.6 ± 4.7 (range 0.5–42) months. Fracture type was assigned based on the postoperative diagnosis of the operative report. Time-to-surgery was defined as the interval between the time of injury and the surgery start time. Demographics were similar among all three age groups as shown in Table 1. The population included 138 (73%) type III SCH fractures and 52 (27%) type IV fractures. There were 26 patients < 2 years old, 111 4–6 years old, and 53  > 8 years old. Duration of pinning and casting were slightly longer in the oldest compared with the middle age group (*p* < 0.004).

**Table 1:** Demographics by age group. (BMI, follow-up, and time-to-surgery are presented as mean values in kg/${\text{m}}^{2}$, months, and hours, respectively)

	Age < 2	Age 4–6	Age > 8
*n*	26	111	53
Male	10	53	35
Female	16	57	18
BMI (kg/m^2^)	18.4	17.6	18.3
Gartland III	18	87	33
Gartland IV	8	24	20
Follow-up (months)	2.4	3.7	3.8
Time-to-surgery (hours)	16.1	14.2	15.9
Time to pins pulled (days)	24.9	23.6	26.1
Duration of casting (days)	25.5	23.8	27.7

Clinical outcomes by age group are presented in Table 2. Age was a significant predictor of nerve palsy on admission. Patients were most likely to present with a nerve palsy in the middle age group and least likely in the youngest age group (< 2 years = 0%; 4–6 years = 25.2%; > 8 years = 20.8%; *p* = 0.006). Increased age was also associated with an increase in mean operative time (< 2 yo = 21.8 min; 4–6 yo = 43.0 min; > 8 yo = 80.7 min; *p* < 0.001) and mean fluoroscopy time (< 2 yo = 22.9 s; 4–6 yo = 59.5 s; > 8 yo = 171.9 s; *p* < 0.001). Elbow stiffness was also more likely to occur in older patients. Full elbow flexion was recorded at final follow-up in 20 (77%) patients < 2 years of age, 73 (66%) in the 4–6 year group, and 23 (43%) in the > 8 year group (*p* = 0.005). Full elbow extension was recorded at final follow-up in 25 (96%) patients in the < 2 year group, 98 (88%) in the 4–6 year group and 34 (64%) in the > 8 year group (*p* < 0.001). Multivariable analysis indicated that stiffness was more likely in older patients even after adjusting for length of follow-up.

**Table 2:** Clinical outcomes compared by age group

	Age < 2	Age 4–6	Age > 8	*p* value
Associated conditions				
Nerve palsy	0.0% (0/26)	25.2% (28/111)	20.8% (11/53)	*0.006*
Compartment syndrome	0.0% (0/26)	4.5% (5/111)	1.9% (1/53)	0.595
Surgical characteristics				
Open reduction	0.0% (0/26)	8.1% (9/111)	13.2% (7/53)	0.149
Surgery duration (mins)	21.8	43	80.7	< *0.001*
Fluoroscopy time (s)	22.9	59.5	171.9	< *0.001*
Complications				
Pin tract infection	3.8% (1/26)	1.8% (2/111)	7.6% (4/53)	0.187
Cubitus varus	0.0% (0/26)	0.9% (1/111)	0.0% (0/53)	> 0.999
Reoperation	0.0% (0/26)	4.5% (5/111)	3.8% (2/53)	0.865
Stiffness in flexion	23.1% (6/26)	34.2% (38/111)	56.6% (30/53)	*0.005*
Stiffness in extension	3.8% (1/26)	11.7% (13/111)	35.9% (19/53)	< *0.001*
Stiffness in either flexion or extension	23.1% (6/26)	37.8% (42/111)	66.0% (35/53)	< *0.001*

Surgery duration and fluoroscopy time are presented as mean values

*p* values with statistical significance are italicized

Older patients were more likely to undergo open reduction, but this difference did not reach statistical significance (< 2 years = 0%; 4–6 years = 8.1%; > 8 years = 13.2%; *p *= 0.149). Age also did not correlate with the incidence of complications including compartment syndrome (*p* = 0.60), pin tract infection (*p* = 0.19), cubitus varus (*p* > 0.999), or reoperation (*p* = 0.87).

## Discussion

This study identified increasing patient age as a predictor of elbow stiffness following operative fixation of SCH fractures. Our findings are consistent with those of Fletcher et al. who reported increased postoperative stiffness in older patients with SCH fractures [6]. The authors also found that older patients were much more likely to have experienced higher energy mechanisms of injury such as falls from height, motor vehicle accidents, or falls from moving objects such as bicycles. This, combined with a higher incidence of open fractures in their study, suggests that older patients may suffer more complications, such as limited postoperative ROM, because they experience worse injuries. These findings may account for some of the differences noted here since older patients may have higher mechanisms of injury and therefore likely have more soft tissue injury [10–12]. Correspondingly, we found that duration of casting and duration of pinning were slightly longer in the older age group compared to the middle age group. At our institution it is standard practice for patients to be casted for 3–4 weeks and at that time have their pins pulled with no additional immobilization. Therefore, this difference was not of clinical significance given that all age groups had duration of casting and pinning within the standard practice timeframe. Furthermore, it is important to note that we observed no difference in the incidence of type IV injuries in each age group (30.8% vs. 21.6% vs. 37.7%, *p* = 0.073).

Morrey et al. defined functional ROM of the elbow for adults to be 30°–130° [13]. Eleven patients in our study had a ROM at final follow-up that fell outside of this functional range. Most of these patients, 72.7% (8/11), were in the oldest age group (> 8 years old). The remainder were from the middle age group (4–6 years old). No patients in the youngest age group in this study had functionally limiting elbow ROM. According to Sardelli et al., modern functional ROM of the elbow may actually be even greater, considering the extensive use of cell phones and computers that Morrey et al. did not include as activities of daily living when they published it in 1981 [14].

Some studies suggest that stiffness following operative fixation of SCH fractures may not be lasting. Zionts et al. described the evolution of return of elbow range of motion following closed reduction percutaneous pinning (CRPP) for SCH fractures [15]. The pattern described by the authors began with sudden but incomplete return of ROM followed by gradual return to full ROM. In their study elbow ROM was 72% of that of the contralateral elbow at 6 weeks postoperatively, 86% at 12 weeks, 94% at 26 weeks, and 98% at 1 year. They also found that improvements in ROM could happen up to 1 year postoperatively. Spencer et al. corroborated these findings of large initial improvements in elbow ROM and later gradual improvements [16]. Our mean overall follow-up of 3.2 months may not have been long enough to capture all eventual improvements in ROM. However, we demonstrate that up to at least 12 weeks postoperatively older patients with SCH fractures may be at greater risk of debilitating elbow stiffness when compared to other age groups.

Although these studies may dispel concerns over permanent elbow stiffness, further investigation is needed to determine the role of postoperative therapy in restoring full elbow ROM [10]. Schmale et al. found no impact of physical therapy on elbow ROM at any time point up to 15 weeks postoperatively, however this study focused solely on closed treatment of SCH fractures [17]. These findings argue against having a low threshold for initiating therapy in older patients to combat anticipated stiffness. While our study did not follow patients out long-term, our findings suggest that older patients might benefit from an earlier referral for physical therapy when compared with younger patients.

Fletcher et al. reported that their older patient cohort was four times more likely to be referred to occupational therapy, which carries significant costs. Even among those with insurance, there is a large variation in the amount of out-of-pocket costs associated with occupational therapy [6]. Co-pays, missed work, and transportation for physical therapy can also be burdensome [18]. In their 2017 analysis of both the direct and indirect costs of treating distal radius fractures, Swart et al. found occupational therapy to be the fifth highest cost associated with operative treatment, after implants and ahead of recovery room, inpatient, and emergency department costs [19]. High costs of therapy can decrease compliance with regimens and increase disability [18].

Additionally, there are financial costs associated with a long period of elbow stiffness that must be considered. The psychological and societal effects of the cosmetic appearance of a stiff elbow must also be considered. Although postoperative therapy may not produce a long-term difference in elbow ROM, it presents the potential to reduce these psychosocial stresses. Further study is needed to assess the utility of postoperative therapy by age.

We analyzed differences in operative time and fluoroscopy time, specifically using fluoroscopy time as a surrogate for difficulty of reduction [20]. Age was a significant predictor of mean operative time (< 2 yo = 21.8 min; 4–6 yo = 43.0 min; > 8 yo = 80.7 min; *p* < 0.001) and mean fluoroscopy time (< 2 yo = 22.9 s; 4–6 yo = 59.5 s; > 8 yo = 171.9 s; *p* < 0.001) (Table 2). Older patients may have fractures that are more difficult to reduce because they have higher energy mechanisms of injury. This may cause increased displacement, soft tissue injury, and swelling. Increasing total operative and fluoroscopic time with age is important to note for the purposes of managing the expectations of the operative team, anesthesiologist, and patient’s family perioperatively.

Overall, our findings can be applied to create a better framework for informing patients and parents on what to expect both intraoperatively and postoperatively following supracondylar humerus fractures.
